# Hypoglycemia is not a defining feature of metabolic crisis in mitochondrial 3‐hydroxy‐3‐methylglutaryl‐CoA synthase deficiency: Further evidence of specific biochemical markers which may aid diagnosis

**DOI:** 10.1002/jmd2.12146

**Published:** 2020-06-30

**Authors:** Tracey A. Conlon, Patricia E. Fitzsimons, Ingrid Borovickova, Fidelma Kirby, Sinéad Murphy, Ina Knerr, Ellen Crushell

**Affiliations:** ^1^ National Centre for Inherited Metabolic Disorders Children's Health Ireland at Temple Street Dublin Ireland; ^2^ School of Medicine University College Dublin Dublin Ireland; ^3^ Department of Paediatric Laboratory Medicine Children's Health Ireland at Temple Street Dublin Ireland; ^4^ Department of Paediatric Intensive Care Children's Health Ireland at Temple Street Dublin Ireland; ^5^ Department of General Paediatrics Children's Health Ireland at Temple Street Dublin Ireland

**Keywords:** 3‐hydroxyglutarate (3HG), 4‐hydroxy‐6‐methyl‐2‐pyrone (4HMP), HMG‐CoA synthase deficiency, hypertriglyceridemia, hypoglycemia, ketogenesis

## Abstract

Mitochondrial 3‐hydroxy‐3‐methylglutaryl‐CoA (HMG Co‐A) synthase (mHS) deficiency is an autosomal recessive disorder of ketone body synthesis which has traditionally been associated with hypoketotic hypoglycemia, hepatomegaly and encephalopathy, presenting in early childhood following a period of fasting. We report the third case of mHS deficiency presenting in the absence of hypoglycemia, with profound biochemical abnormalities and further evidence of potential specific diagnostic biomarkers. A previously well, 20‐month old, unvaccinated male, of nonconsanguineous Polish heritage, presented with encephalopathy, hepatomegaly, severe metabolic acidosis, and mild hyperammonemia following a brief intercurrent illness. The patient was reported to have taken colloidal silver prior to presentation, posing a further diagnostic challenge. Additionally, he developed features suggestive of hemophagocytic lymphohistiocytosis during treatment. While the patient was normoglycemic prior to dextrose administration, the sample was markedly lipemic, with significant hypertriglyceridemia detected. Urine organic acid analysis revealed dicarboxylic aciduria with 4‐hydroxy‐6‐methyl‐2‐pyrone (4HMP) and the presence of three other previously reported putative biomarkers for mHS deficiency. Glutarate was markedly elevated in the initial chromatogram, with a mild increase in 3‐hydroxyglutarate (3HG) persisting. Raised acetylcarnitine was detected on acylcarnitine profile. Molecular genetic analysis of the *HMGCS2* gene identified compound heterozygosity for known pathogenic mutations c.634G>A and c.1016+1G>A, confirming the diagnosis of mHS deficiency. This case provides further evidence that hypoglycemia is not invariably present in symptomatic mHS deficiency. We propose that elevated acetylcarnitine, triglycerides, and 3HG are additional biochemical features during acute presentations. With the expansion of novel biomarkers, further cases of this rare disorder may emerge.

**SYNOPSIS**

Mitochondrial 3‐hydroxy‐3‐methylglutaryl‐CoA (HMG‐CoA) synthase (mHS) deficiency is a rare, potentially life‐threatening disorder, which should be considered in the patients presenting with encephalopathy, hepatomegaly and acidosis, even in the presence of marked ketonuria and without hypoglycemia.

## 1 INTRODUCTION

Mitochondrial 3‐hydroxy‐3‐methylglutaryl‐CoA (HMG‐CoA) synthase (EC 2.3.3.10) catalyzes the first and rate‐limiting step of ketone body biosynthesis from fatty acids and is essential for providing energy to the brain during fasting.^1^, ^2^ HMG‐CoA synthase deficiency (mHS; OMIM #605911) is a rare, autosomal recessive disorder of metabolism, typically presenting with severe hypoketotic hypoglycemia following a prolonged fasting period or intercurrent illness. This may be accompanied by vomiting, hepatomegaly, lethargy, and potentially life‐threatening encephalopathy. Clinical presentation is highly variable, and the diagnosis is often impeded by nonspecific clinical signs and variable biochemical findings.

First described over 20 years ago,^3^ 29 patients have now been identified with gene mutations in *HMGCS2* (GenBank NM_005518.2), which encodes the mitochondrial HMG Co‐A synthase enzyme, with varying phenotypes of mHS deficiency described. Recent case reports^4^, ^5^, ^6^, ^7^ have expanded our knowledge greatly and provided insight into the clinical and biochemical diversity of this disorder. This report provides further evidence of the variety of phenotypic manifestations associated with mHS deficiency, provides further evidence for previously reported putative biomarkers and suggests new biochemical markers which may aid future diagnosis.

## 2 MATERIALS AND METHODS

### 2.1 Biochemical analysis

Urinary organic acid levels were analyzed using gas chromatography mass spectrometry (GC/MS; 5975‐6890N Agilent Technologies, Santa Clara, California) at Children's Health Ireland at Temple Street, after ethylacetate/diethyl ether solvent extraction and trimethylsilyl derivatization of urine samples using heptadecanoate and heptanoyl glycine internal standards as previously described.^8^, ^9^, ^10^, ^11^

The dried blood spot acylcarnitine profile was analyzed using tandem mass spectrometry (MS/MS) (Quattro Premier, Waters Micromass, Milford, Massachusetts) at Children's Health Ireland at Temple Street, after butyl‐derivatization of samples as previously described.^12^

### 2.2 Genetic analysis

Sequence analysis of the nine coding exons (including flanking sequences) of the *HMGCS2* gene was performed by MVZ Dr. Eberhard & Partner Dortmund, Laboratoriumsmedizin, Dortmund, Germany, on a peripheral EDTA blood sample. Deletion/duplication analysis: MLPA, MRC‐Holland, Kit P068‐B2. The nucleotides and codons are numbered according to HGVS (Ref.‐Seq. NM_005518.3).

## 3 CASE PRESENTATION

The second male child of unrelated Polish parents, presented at 20 months of age to a regional unit, with profound encephalopathy, following 2 days of vomiting with associated limited oral intake. He was a previously well child, with normal growth and development. He had previously coped well with minor illnesses and had a normal diet. There had been no perinatal concerns. At the time of presentation, it was reported he had been taking a variety of herbal and other alternative medical preparations, including colloidal silver. He was unvaccinated by parental choice. There was no relevant family history.

Clinical examination at presentation was remarkable for severe encephalopathy with a Glasgow Coma Scale score of 4/15, poor perfusion, Kussmaul's breathing and hepatomegaly. Initial investigations indicated normoglycemia (blood glucose 4.6 mmol/L, reference range 3.3‐5.6), with a severe high anion‐gap metabolic acidosis (pH 6.8, reference range 7.28‐7.4; HCO3‐1.3 mmol/L, reference range 20‐27; anion gap 37 mmol/L, reference range 8‐16). Serum lactate (1.1 mmol/L, reference range 0.6‐2.4), CK (48 U/L, reference range 20‐155), glucose, and ketone bodies remained normal. Ammonia was mildly elevated at 159 μmol/L (reference range 0‐65), as were transaminases (AST 183 U/L, reference range 0‐50; ALT 354 U/L reference range 0‐45). Serum white cell count was elevated at 21.0 × 10^9^/L (reference range 5‐15) with neutrophilia (16.87 × 10^9^/L, reference range 1‐8.5) and anemia (hemoglobin 78 g/L, reference range 105‐135). Samples were strikingly lipemic, with no chylomicronemia post centrifugation. Serum triglycerides were found to be markedly elevated at 34 mmol/L (reference range 0‐0.85) with hypercholesterolemia (total cholesterol 5.8 mmol/L, reference range 2‐4.4), but decreased high density lipoprotein cholesterol (0.24 mmol/L, reference range 1.2‐2.4). Serum amylase was normal. No cutaneous xanthomas were present.

He was intubated, ventilated, and transferred to a tertiary center for intensive management. He was commenced on intravenous fluids including dextrose 10% at presentation (following initial sampling). Broad spectrum antibiotics, antiviral medication, and bicarbonate infusion were commenced. Upon transfer, central venous access was secured and dextrose concentration maximized (glucose infusion rate 8.7 mg/kg/min). Further samples were taken in consideration of an underlying inborn error of metabolism. Advice was sought from The National Poisons Information Centre, with regards to the history of ingestion of colloidal silver and various other unspecified herbal preparations. Toxicology screen was negative and no specific therapy was instituted.

Biochemical parameters recovered gradually, with resolution of metabolic acidosis at 13 hours post presentation. Hypertriglyceridemia had significantly improved at day 3 (triglyceride 1.24 mmol/L, reference range 0‐0.85). He had a prolonged admission, as there was additional concern for a secondary diagnosis of hemophagocytic lymphohistiocytosis (HLH). He developed persistent pyrexia with leukopenia, thrombocytopenia, anemia, and an elevated ferritin and LDH following metabolic recovery. He underwent extensive serology and virology studies with Epstein Barr virus (EBV) (polymerase chain reaction and IgM), adenovirus and rhinovirus positivity. Subsequent immunological studies including bone marrow aspirate, bone marrow biopsy, and extended lymphocyte subsets were normal.

An abdominal ultrasound showed marked hepatomegaly with a coarse echotexture and increased echogenicity, suggestive of fatty infiltration. Computed tomography brain (noncontrast) on the day of presentation was normal. Magnetic resonance imaging brain (T1, T2, T2 flair, and DWI sequences) at day 13 identified prominent cerebral sulci suggestive of mildly reduced parenchymal volume, with normal parenchymal signal and otherwise no diagnostic abnormality.

The patient made a full clinical recovery, was extubated on day 3 and had a normal neurological examination on day 4. He required intensive care support for 4 days and inpatient care for 23 days. Hepatomegaly and transaminitis were slow to recover (complete resolution at day 28) and were attributed to the secondary diagnosis of EBV infection. Following discharge, fasting times were limited and vaccination recommended. At 30 months follow‐up, there has been no recurrence of metabolic crisis with intercurrent illness. Growth and development have been normal to date and he remains unvaccinated.

## 4 RESULTS

**FIGURE 1:**
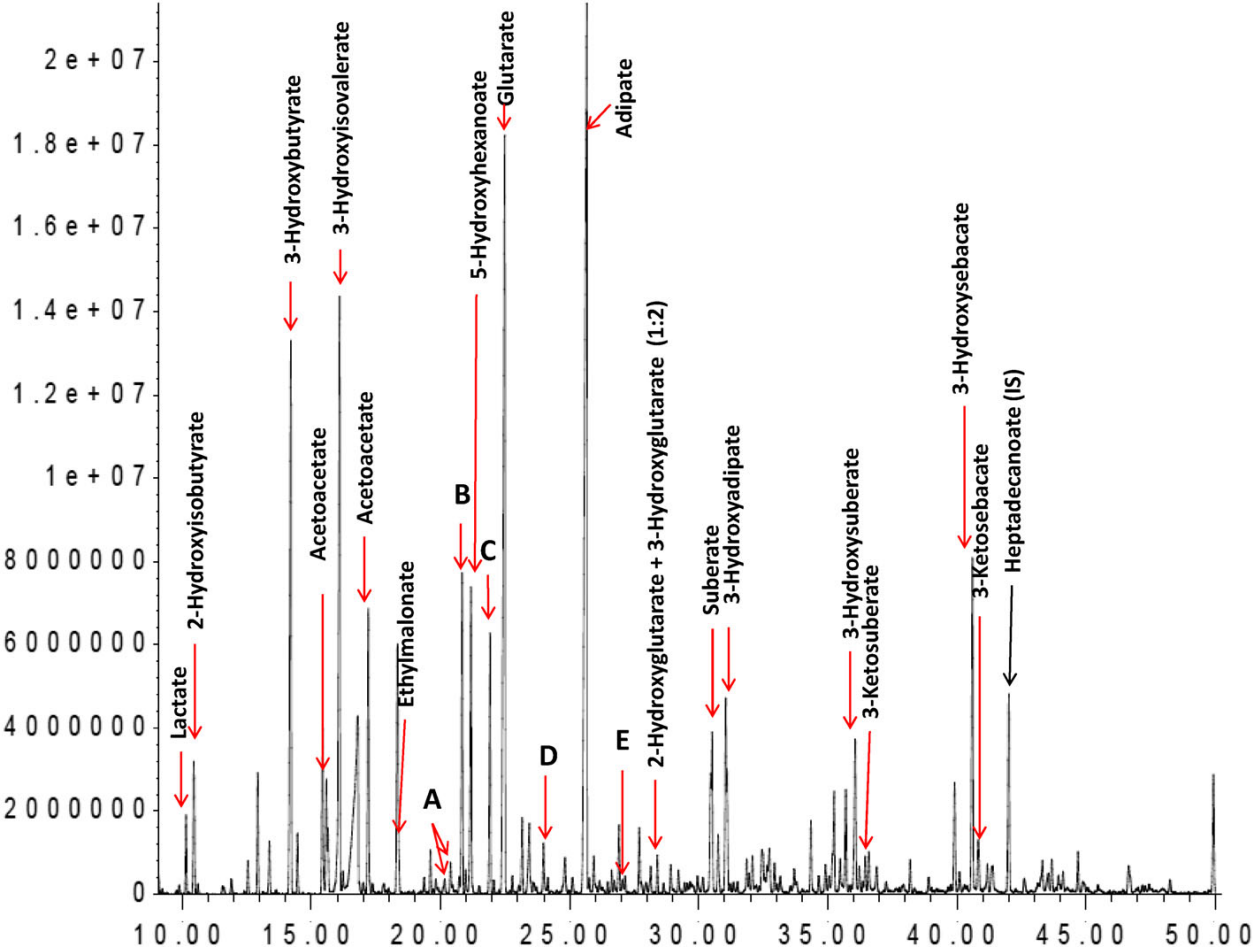
On presentation: Marked ketonuria. Inappropriate dicarboxylic aciduria. Elevated 3‐HIVA, 5HHEX, glutarate, 3HG, and biomarkers of mHS (A‐E). As 2‐ and 3‐hydroxyglutarate co‐elute on our qualitative analysis, the ratios of 2‐ and 3‐hydroxyglutarate was determined by comparing specific extracted ions m/z 85, 157 for 2‐hydroxyglutarate and m/z 185, 259 for 3‐hydroxyglutarate

**FIGURE 2:**
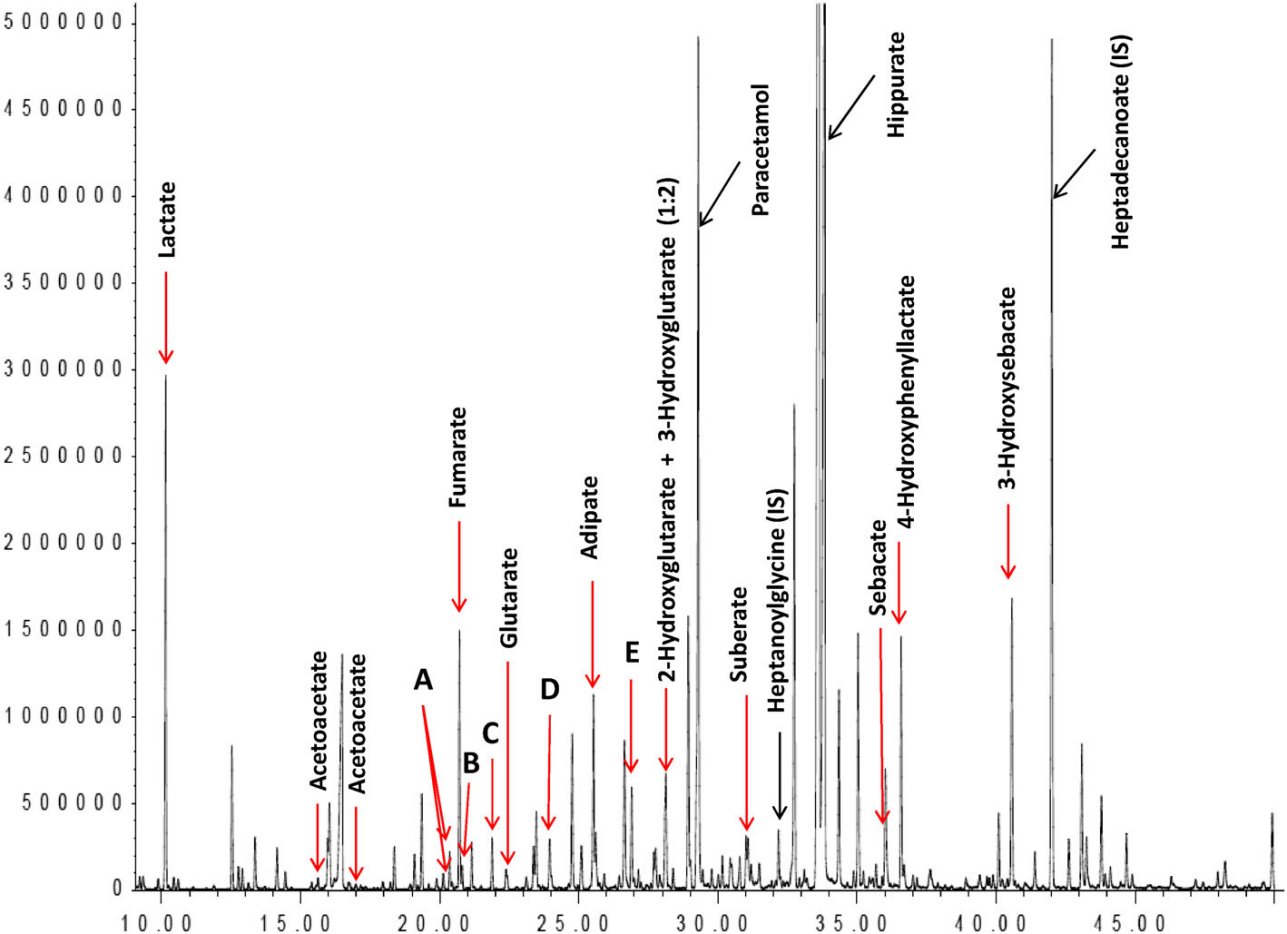
Post treatment: Mild increase in lactate and TCA cycle intermediates. Mild increase in dicarboxylic acids with no ketone bodies detected and disproportionate increases in 3HG, 4HMP (D) and other biomarkers of mHS deficiency (A, B, C, E)

Urine organic acid analysis at presentation revealed an inappropriate pattern of relative hypoketotic dicarboxylic aciduria, suggestive of a fatty acid oxidation disorder. There was a notable increase in medium chain 3‐oxodicarboxylic acids, 3‐hydroxyisovalerate (3HIVA), glutarate and disproportionate increases in 5‐hydroxyhexanoate (5HHEX) and 3‐hydroxyglutarate (3HG). In the initial sample (Figure 1), excretion of 4‐hydroxy‐6‐methyl‐2‐pyrone (4HMP) (D) was detected, though less than ketone bodies. Acidosis and lipemia gradually corrected and repeat urine organic acid analysis normalized (by day three of treatment), with the exception of persistence of 4HMP and 3HG, which were disproportionate to ketone bodies (Figure 2). 3HG was no longer detectable on repeat urine organic acid analysis at day 13. 4HMP was identified on two subsequent samples at day 20 and at 1 year follow‐up. The first sample was retrospectively examined and trans‐3‐hydroxy‐hex‐4‐enoate (A) 3,5‐dihydroxyhexanoate 1,5 lactone (B), trans‐5‐hydroxyhex‐2‐enoate (C), 3,5‐dihydroxyhexanoate (E), previously reported biomarkers of mHS deficiency were present.

Acylcarnitine analysis showed a relative increase in acetylcarnitine (C2) (48.1 μmol/L, reference range 5.5‐38), also previously reported with mHS deficiency. Free carnitine was normal (16.2 μmol/L, reference range 15.5‐46.7). C2/C0 ratio was elevated (2.96).

Molecular genetic analysis of the *HMGCS2* gene in leukocyte DNA from the patient, identified compound heterozygosity for a missense mutation c.634G>A (p.G212R)^13^ and a splice mutation c.1016+1G>A (IVSS+1g>a),^14^ which have been previously reported as pathogenic mutations associated with mHS deficiency. Compound heterozygosity for these mutations has been identified in one other patient with this disorder.^14^ Parental samples were subsequently obtained, with each parent harboring one pathogenic variant each.

## 5 DISCUSSION

Since the first description of this rare, treatable disorder, our knowledge of its clinical, biochemical and genetic diversity has greatly expanded. This case of mHS deficiency, which despite having many similarities to previously published cases, is only the third case known to have presented without hypoglycemia. The mechanism by which hypoglycemia was averted remains unclear. Similar to a recent Japanese case in which hypoglycemia was not detected and the patient was positive for influenza virus,^5^ our case had a persistent high grade fever and was positive for EBV, adenovirus and rhinovirus. The only other case^4^ in which normoglycemia occurred in the acute phase was investigated following the administration of intravenous fluids. The severity of both the clinical presentation and the other biochemical abnormalities in both our case and the Japanese case,^5^ suggest it is not related to enzyme activity or a less severe crisis. It is highly probable that further cases of mHS deficiency may be detected if one considers the possibility of its presentation in the absence of hypoglycemia. Future research is needed to determine the etiology of hypoglycemia avoidance in these cases.

Hepatomegaly/fatty liver has been identified during metabolic decompensation in 17 patients (including this report) with mHS deficiency,^3^, ^4^, ^5^, ^6^, ^14^, ^15^, ^16^, ^17^, ^18^, ^19^ but not all.^6^, ^7^ It has been reported in cases with and without hypoglycemia, to varying degrees, likely reflecting the extent of ketogenic stress. Our case presented with marked hepatomegaly with fatty infiltration and associated elevated liver enzymes in the acute phase which fully recovered by day 28.

The timely diagnosis of mHS deficiency is not only hampered by its nonspecific clinical presentation, but additionally by the lack of unique biochemical markers and need for thorough investigation during the acute phase. The presence of significant lipemia in our initial samples and identification of significant hypertriglyceridemia complicated our patient's diagnosis. Hypertriglyceridemia and normoglycemia has been observed in one other patient with mHS deficiency.^4^ In both cases, and in several other reported cases of mHS deficiency, a high anion‐gap acidosis was observed when the patients were acutely symptomatic.

The urine organic acid profile in our patient showed similarities to those previously published, in addition to new potential biomarkers. The dominant picture was that of secondary products of fatty acid oxidation, with 3‐hydroxydicarboxylic aciduria and ketonuria with an inappropriate adipate/3‐hydroxybutyrate ratio, as previously reported in the literature.^3^, ^5^, ^6^, ^14^, ^15^, ^16^, ^17^ Glutarate was markedly increased and there were disproportionate increases in 3HG and 4HMP. 4HMP was initially identified as a putative biomarker for decompensated mHS deficiency in five patients^6^ and has now been detected in four further cases,^4^, ^5^, ^7^ including this report. Of interest, 4HMP remained detectable at 1 year follow‐up, while our patient was well. This may be of further diagnostic utility in future cases.

Three other abnormal metabolites suggested to be characteristic following the detailed report by Pitt et al were additionally identified in our proband; 3,5‐dihydroxyhexanoic 1,5 lactone, trans‐5‐hydroxyhex‐2‐enoate, and 3,5‐dihydroxyhexanoate. We have also noted these markers (including 4HMP) in severely ketotic patients and thus these metabolites are not pathognomonic for this condition. Interestingly, a persistent increase in 3HG was noted even following the correction of acidosis in our patient. This has not previously been described in any patients with mHS deficiency and may be a future biomarker for the disorder.

This child presented normoglycemic, with profound encephalopathy, hyperammonemia and subsequently had a normal neurological examination, with essentially normal neuroimaging. In patients with mHS deficiency, we do not see, for example, the typical striatal injury one can expect in patients with acute encephalopathy due to glutaric aciduria type 1. However, given the severity of the comatose state in our case the presence of 3HG may have been a contributing factor to his clinical presentation, as 3HG may cause a disruption of human endothelial cell function and hence the blood brain barrier.^20^ He had marked ketonuria at the time of presentation, which has been previously reported and may result from HMG‐CoA formation from leucine metabolism. We hypothesize that the extent of ketonuria in this case may have been cerebroprotective. It is important to note that the presence of ketonuria does not exclude a diagnosis of a disorder of ketogenesis.

In conclusion, mHS is a rare disorder, but is likely underdiagnosed. Here, we describe a complex case of an initial presentation of mHS deficiency, which was complicated by herbal medicine ingestion and concern for HLH syndrome in an unvaccinated child. We have identified elevated triglycerides, with low HDL cholesterol and elevated 3HG as additional biochemical features of this disorder. Additionally, this new case provides further evidence for 4HMP as a specific diagnostic biomarker, which remained detectable at 1 year follow‐up.

## CONFLICT OF INTEREST

Tracey A. Conlon, Patricia E. Fitzsimons, Ingrid Borovickova, Fidelma Kirby, Sinéad Murphy, Ina Knerr, and Ellen Crushell declare that they have no conflicts of interest pertaining to the manuscript.

## AUTHOR CONTRIBUTIONS

Tracey A. Conlon, Fidelma Kirby, Sinéad Murphy, Ina Knerr, and Ellen Crushell: Clinicians from various pediatric specialties who provided care to the patient, contributed significantly to writing the manuscript, and critically reviewed the manuscript.

Patricia E. Fitzsimons and Ingrid Borovickova: Were essential in the analysis, diagnosis and interpretation of the molecular and biochemical lab results and critically reviewed and edited the manuscript.

## INFORMED CONSENT

All procedures followed were in accordance with the ethical standards of the responsible committee on human experimentation (institutional and national) and with the Helsinki Declaration of 1975, as revised in 2000 (5).

Informed consent for publication of this manuscript was obtained from the patient's parents.
